# Brainstem Stroke and Dysphagia Treatment: A Narrative Review on the Role of Neuromodulation, Skill-Based Swallowing Training and Transient Receptor Potential Agonists

**DOI:** 10.3390/audiolres15060156

**Published:** 2025-11-12

**Authors:** Ivy Cheng, Wan-Qi Li, Shaheen Hamdy, Emilia Michou, Maggie-Lee Huckabee, Noemí Tomsen, Pere Clavé, Rainer Dziewas

**Affiliations:** 1 Academic Unit of Human Communication, Learning and Development, Faculty of Education, University of Hong Kong, Hong Kong; shaheen.hamdy@manchester.ac.uk; 2 Division of Diabetes, Endocrinology and Gastroenterology, School of Medical Sciences, Centre for Gastrointestinal Sciences, Faculty of Biology, Medicine and Health, University of Manchester, Manchester M3 9NT, UK; wanqi.li@manchester.ac.uk; 3 Department of Rehabilitation Medicine, Guangzhou First People’s Hospital, Guangzhou Medical University, Guangzhou 510180, China; 4 Department of Speech Language Therapy, School of Health Rehabilitation Sciences, University of Patras, GR26504 Patras, Greece; emiliamichou@upatras.gr; 5 Rose Centre for Stroke Recovery and Research, School of Psychology Speech and Hearing, University of Canterbury, Christchurch 8140, New Zealand; maggie-lee.huckabee@canterbury.ac.nz; 6 Gastrointestinal Physiology Laboratory, Hospital de Mataró, Universitat Autònoma de Barcelona, 08304 Mataro, Spain; ntomsen@csdm.cat (N.T.); pere.clave@ciberehd.org (P.C.); 7 Centro de Investigación Biomédica en Red en Enfermedades Hepáticas y Digestivas (CIBEREHD), Instituto de Salud Carlos III, 28029 Madrid, Spain; 8 Department of Neurology and Neurorehabilitation, Klinikum Osnabrück (Academic Teaching Hospital of the University of Münster), 49076 Osnabrueck, Germany; rainer.dziewas@klinikum-os.de; 9 Department of Neurology, University Hospital Münster, 48149 Muenster, Germany

**Keywords:** brainstem, dysphagia, neuromodulation, skill training, stroke, transient receptor potential agonists

## Abstract

Swallowing is mediated by the central nervous system, including cortical and subcortical structures, the cerebellum, and the brainstem. The brainstem contains the swallowing centre that is crucial for initiating and coordinating swallowing. Consequently, brainstem damage due to stroke often leads to severe and persistent dysphagia. The aim of the present narrative review is to provide an overview of dysphagia following brainstem stroke and its management. It summarizes the physiology and pathophysiology of dysphagia following brainstem stroke and the available therapeutic options, and evaluate their effectiveness for dysphagia following brainstem stroke, which would promote the development of therapeutic protocols. Neuromodulatory techniques, including pharyngeal electrical stimulation (PES), repetitive transcranial magnetic stimulation (rTMS), and transcranial direct current stimulation (tDCS), modulate the excitability of corticobulbar circuits. These techniques promote neuroplasticity through peripheral or cortical electrical or electromagnetic inputs. Skill-based swallowing training emphasizes cortical involvement in enhancing swallowing skill, offering a targeted approach to behavioural rehabilitation. Finally, transient receptor potential (TRP) agonists increase sensory inputs to the swallowing system by stimulating the sensory receptors in the oropharynx, potentially activating the swallowing network. While these options have shown promise in dysphagia rehabilitation following stroke, most the available data comes from patients with mixed stroke lesions, with limited data focused specifically on brainstem lesions. Therefore, the evidence for their efficacy in patients with brainstem stroke remains underexplored. Therefore, treatment decisions should rely on the understanding of swallowing physiology, neuroplasticity, and clinical evidence from related stroke populations.

## 1. Introduction

The brainstem is a complex structure that includes sensorimotor neural pathways and the swallowing central pattern generator (CPG). Its vital role has been demonstrated both in studies of healthy individuals and in clinical research on dysphagia. Brainstem stroke often results in severe and persistent dysphagia, leading to life-threatening consequences such as aspiration pneumonia, malnutrition and prolonged hospitalization due to dysphagia-related complications. Apart from clinical consequences, dysphagia has significant psychosocial impacts, as social embarrassment from choking during meals can cause anxiety and isolation. Furthermore, it contributes to increased healthcare cost due to the complex and prolonged recovery process. Therefore, there is a need for effective treatment to manage dysphagia in patients with brainstem stroke. This narrative review summarizes the physiology and pathophysiology of dysphagia resulting from brainstem stroke, reviews the current evidence on dysphagia treatments, and discusses the challenges in managing dysphagia in patients with brainstem stroke.

## 2. Physiology and Pathophysiology of Dysphagia After Brainstem Stroke

### 2.1. Swallowing and Brainstem Anatomy

Swallowing is a highly coordinated activity involving three stages: oral, pharyngeal, and esophageal. While the oral stage is voluntary, the pharyngeal and esophageal phases rely heavily on brainstem control [1]. Within the brainstem, a network of nuclei and neural pathways work in synchrony to ensure the safe and efficient transit of food of any consistency, saliva, and medications, from the oral cavity to the stomach [2].

**Figure 1:**
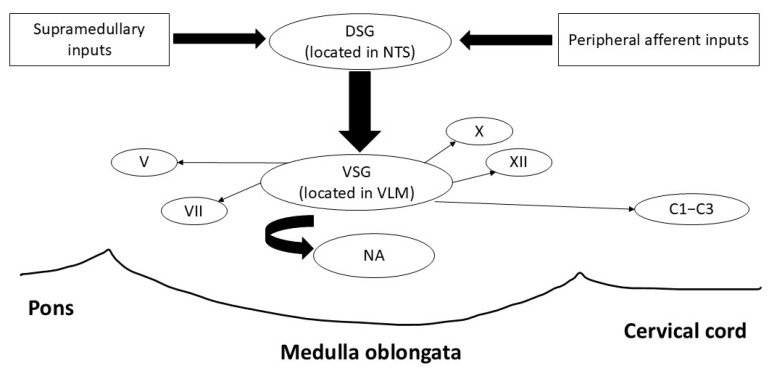
Schematic representation of the organization of the swallowing central pattern generator (CPG) located in the brainstem. The CPG comprises two groups of neurons that can be categorized into the dorsal swallowing group (DSG) and the ventral swallowing group (VSG). Neurons in the DSG receive inputs from peripheral receptors and supramedullary structures and activate the VSG neurons. The VSG neurons then send signals to the motor nuclei. Adapted from Jean et al. (2001) [7].

The brainstem is composed of the midbrain, the pons, and the medulla oblongata, located in the posterior part of the brain, acting as a conduit between the cerebrum, cerebellum, and spinal cord [3]. Key anatomical structures include the nucleus tractus solitarius (NTS) and the nucleus ambiguus (NA), both located in the medulla (Figure 1). The NTS functions as a sensory hub, integrating input from cranial nerves V, VII, IX, and X to detect bolus characteristics such as texture, volume, and position [4]. This sensory information is critical for triggering and fine-tuning the swallowing reflex. Moreover, the NTS also plays a key role in regulating autonomic functions through synaptic connections with cortical, subcortical and cerebellar regions [5]. The NA serves as the primary motor nucleus for the pharyngeal, laryngeal and upper esophageal muscles, coordinating their movements through motor output via cranial nerves IX, which primarily provides motor innervation to the muscles of the pharynx, and X. This activity ensures effective bolus propulsion and airway protection, which are vital for safe swallowing. Additional structures, such as the hypoglossal nucleus and dorsal motor nucleus of the vagus nerve, contribute to tongue movement and esophageal peristalsis, while the spinal trigeminal nucleus provides supplementary sensory information (Table 1). In addition to the NA, the motor activity during swallowing is also modulated by higher brain centres such as the primary motor cortex [6].

The motor nuclei (V, VII, IX, X and XII), along with two main groups of interneurons—the dorsal swallowing group (DSG) in the NTS and adjacent reticular formation and the ventral swallowing group (VSG) in the ventrolateral medulla above the NA—form a complex unit called the CPG [2,7]. The DSG comprises generator interneurons that are involved in triggering and sequencing of motor events, whereas the VSG comprises switching interneurons and acts as a relay unit in which neurons are activated by DSG neurons, and then sends signals to motoneurons in motor nuclei that innervates the muscles in the oropharynx and esophagus [7]. The CPG serves a dual role in regulating both respiration and swallowing—two temporally coordinated processes essential for airway protection. During swallowing, the CPG induces a transient inhibition of the respiratory rhythm (swallowing apnoea) to prevent airway penetration. Studies have identified several distinct patterns and temporal relationships between pharyngeal swallow and swallowing apnoea, with initiation of swallow activity during expiratory phase of the respiratory cycle being the most common [8], and such patterns may change with advanced age [9]. Damage to CPG may disrupt airway-deglutitive coordination, which often contributes to impaired swallowing safety and increasing risk of aspiration [10]. Notably, much of the understanding of brainstem function for swallowing comes from the animal literature—using microelectrodes to identify interneurons responsible for swallowing in anesthetized or awake animals [6].

### 2.2. Pathophysiology of Dysphagia After Brainstem Stroke

It is well-recognized that damage to the brainstem can result in dysphagia and usually lead to more severe dysphagia than cortical lesions [6,11]. The relationship between lesion location and dysphagia is of significant interest, as it provides insights into the functional roles of affected regions in swallowing and may aid in predicting dysphagia incidence and recovery outcomes. A meta-analysis of 17 studies involving magnetic resonance imaging (MRI) data reported that lesions in the pons and medial and lateral medulla were highly associated with the presence of dysphagia [12], with highest incidence in lesions in lateral medulla, followed by pons, medial medulla and then midbrain. A functional MRI (fMRI) study observing brain activation during voluntary swallowing found that the brainstem and putamen specifically control laryngeal movement [13]. Another study reported that brainstem infarction is associated with reduced laryngeal elevation and residues in valleculae and pyriform sinus, which could result in ineffective airway protection and bolus clearance, leading to aspiration [14]. A retrospective study using MRI/computerized topography (CT) to investigate dysphagia and brain lesion localization reported a significantly higher rate of enteral tube feeding, history of pneumonia and voice change after swallowing in lesions located in pons and medulla [15].

Medullary strokes are especially devastating because they disrupt both sensory input and motor output at their origin [15]. Damage to the NTS impairs the sensory feedback necessary for initiating and modulating the swallowing reflex, while injury to the NA weakens motor output necessary for swallowing execution, leading to ineffective bolus clearance and reduced airway protection. This dual disruption often results in severe, persistent dysphagia, characterized by delayed or absent swallowing reflexes, aspiration, and a high risk of pneumonia. Lateral medullary syndrome, also known as Wallenberg syndrome, is a notable example of brainstem stroke resulting in dysphagia. Typically caused by occlusion of the posterior inferior cerebellar artery, this syndrome affects both the NTS and NA, leading to sensory deficits and motor dysfunction that severely impair pharyngeal clearance and airway protection [16,17]. Pontine strokes, although less frequently associated with profound dysphagia, can impair motor coordination by disrupting the communication between the cortex and medullary swallowing centres, leading to delayed swallowing reflexes and poor bolus propulsion [18]. Midbrain strokes, again, while less commonly associated with dysphagia, can indirectly affect swallowing by impairing arousal and voluntary initiation of the swallowing process.

Variability in dysphagia severity is influenced by the localization and size of the brain lesion, as well as whether the brainstem lesion is unilateral or bilateral [15,19,20,21]. Given that the structures involved in swallowing are predominantly bilaterally innervated, unilateral damage may produce partial dysphagia, with some preserved function on the unaffected side, while bilateral lesions are usually catastrophic and ultimately non-survivable [22,23,24]. This profound dysfunction frequently necessitates long-term enteral nutrition, such as nasogastric tube feeding or gastrostomy, to prevent aspiration and malnutrition. Moreover, lesion size is one of many factors that can adversely affect outcomes and recovery after stroke; the more extensive the damage to crucial brain regions, the greater the likelihood of dysphagia [21]. The corticobulbar pathways, which descend from the cortex to the brainstem, also play a role in swallowing. Strokes affecting these pathways through discreet lesions in the brainstem circuitry, can result in spasticity and incoordination of the swallowing muscles, further compounding the difficulties faced by patients with brainstem damage [25].

The clinical consequences of dysphagia following brainstem stroke are profound and multifaceted [3,20]. It can lead to life-threatening conditions such as aspiration pneumonia, malnutrition and dehydration, which in turn exacerbate recovery and increase the risk of secondary infections. Psychosocially, dysphagia can be distressing, as fear of choking and social embarrassment during meals often leads to isolation and depression [26]. Dysphagia can also significantly increase healthcare costs due to prolonged hospitalizations and rehabilitation programmes, and the need for permanent nutritional support [27]. Given these severe consequences following brainstem stroke, research has explored the therapeutic values of novel intervention for these patients. In the following sections evaluate the current evidence for neuromodulation, behavioural and pharmacological approaches for dysphagia following brainstem stroke.

## 3. Neuromodulation for Dysphagia After Brainstem Stroke

Neuromodulatory techniques, including peripheral (pharyngeal electrical stimulation [PES]) and central (repetitive transcranial magnetic stimulation [rTMS], transcranial direct current stimulation [tDCS]) approaches, can facilitate recovery from post-stroke dysphagia by promoting neuroplasticity [28,29,30,31]. In patients with brainstem stroke where the CPG is damaged, neuromodulatory techniques may facilitate recovery by stimulating and recruiting residual swallowing-related neural networks.

### 3.1. Pharyngeal Electrical Stimulation (PES)

Pharyngeal electrical stimulation (PES) delivers electrical stimulation to the pharyngeal mucosa via an intraluminal catheter with bipolar ring electrodes [28]. It has been approved by the Food and Drug Administration (FDA) and the European Commission (EC) as a dysphagia treatment. Although PES is a peripheral stimulation, its neuromodulatory effects are centrally driven, as evidenced by changes observed in both central and peripheral neural networks. Early physiological studies demonstrated that PES increases the excitability and representation of the pharyngeal motor cortex [28,32,33], reverses effects of a rTMS-induced “virtual lesion” of the pharyngeal motor cortex [34], enhances bilateral sensorimotor cortical activation [35], and increases saliva level of substance P, a neuropeptide associated with cough and swallow reflexes [36,37].

In stroke patients, meta-analyses of randomized controlled trials (RCTs) suggested that PES is beneficial for post-stroke dysphagia [38]. PES can reduce the risk of penetration and aspiration and improve swallowing function in stroke patients [33,34,39,40,41,42]. Importantly, in tracheotomised stroke patients with severe dysphagia, PES facilitates early decannulation by improving swallowing function and secretion management [40,42]. A recent RCT found that PES could enhance postextubation dysphagia recovery, reduced tube dependency and pneumonia, and shortened hospital stay in acute stroke patients [43,44].

The effects of PES for patients with brainstem stroke has not been studied in detail. Cheng et al. [45] analyzed the factors affecting PES treatment outcomes using data of 98 patients with post-stroke dysphagia with mixed stroke lesions who required mechanical ventilation and tracheotomy from an observational study [46]. They found that among patients who received PES while tracheotomised, those with supratentorial stroke may have better outcomes compared to those who had infratentorial (predominantly brainstem) stroke. It is suggested that although the neuroplastic changes induced by PES may occur at the cortical level, if the brainstem is severely damage, the descending signals may not reach the swallowing muscles for functional improvement. Nonetheless, Bath et al. found that stroke patients with tracheotomy and mechanical ventilation responded well to PES and showed reduction in dysphagia severity and risks of penetration and aspiration, regardless of whether they had supratentorial or infratentorial stroke [46]. A recent case study reported a 53-year-old woman with severe dysphagia following ischemic brainstem and cerebellar stroke who benefited from prolonged PES [47]. She showed improvement in pharyngeal sensation and oral secretion management following 11 sessions of PES, providing further evidence that PES may be beneficial in patients with supratentorial stroke.

### 3.2. Repetitive Transcranial Magnetic Stimulation (rTMS)

Repetitive transcranial magnetic stimulation (rTMS) is a form of non-invasive brain stimulation (NIBS) technique that can enhance cortical excitability and induce neuroplasticity not only in the stimulated region, but also throughout the swallowing-related neural networks via interhemispheric and cerebellar connections, which is particularly relevant when the primary lesion is in the brainstem. The rationale for rTMS in brainstem stroke stems from its capacity to modulate residual cortical and cerebellar networks that interface with bulbar circuits. Even in the presence of medullary lesions, the corticobulbar and corticocerebellar pathways can be harnessed to influence the swallowing CPG indirectly [1]. Stimulation of the pharyngeal motor cortex, particularly on the unaffected hemisphere, has been shown to increase excitability and motor output to the swallowing musculature [29]. Furthermore, cerebellar rTMS has demonstrated downstream modulation of both cortical and bulbar centres, likely via dentatothalamocortical and fastigial projections [48].

Recent studies have demonstrated the feasibility and potential of cerebellar and cortical rTMS to facilitate swallowing recovery after brainstem stroke (Table 2). A meta-analysis by Wang et al. [49] found that patients with brainstem stroke may benefit more from rTMS than those with hemispheric lesions (standardized mean difference [SMD] = 1.53). This is likely due to the strategic targeting of intact cortical pathways to compensate for damaged bulbar circuits. Dong et al. [50] conducted a controlled trial involving 36 patients with medullary or pontine infarcts and showed that bilateral high-frequency (10 Hz) cerebellar rTMS significantly improved swallowing outcomes measured by Penetration Aspiration Scale (PAS) [51], Functional Dysphagia Scale (FDS) [52], and increased cortical motor evoked potential (MEP) amplitudes, although gains in MEP excitability were not linearly correlated with clinical recovery. These results support the role of cerebellum in modulating cortical and brainstem activity involved in swallowing [48]. Sasegbon et al. [53] showed in healthy subjects that 10 Hz cerebellar rTMS could reverse cortical inhibition induced by a virtual lesion, indirectly supporting its therapeutic potential.

In support of these findings, Dai et al. [57] conducted a single-blinded RCT involving 42 patients with subacute infratentorial stroke and dysphagia. The study compared bilateral cerebellar rTMS (biCRB-rTMS) and unilateral (uniCRB-rTMS) with a sham stimulation control. Both active rTMS protocols significantly improved swallowing outcomes measured by Functional Oral Intake Scale (FOIS) [64], Dysphagia Outcome and Severity Scale (DOSS) [65] and PAS, with the bilateral approach showing more consistent functional benefits. However, there were no significant differences in neurophysiological MEP changes across groups, suggesting that functional improvements may not be solely mediated by corticospinal excitability changes [57].

Apart from cerebellar rTMS, other studies have also explored the effects of vagus nerve stimulation and cortical rTMS for dysphagia after brainstem stroke. Lin et al. [56] reported the feasibility of vagus nerve magnetic modulation over a 10-day period, showing marked functional gains in swallowing recovery. Verin et al. (2016) documented a complete restoration of oral feeding in two chronic lateral medullary syndrome cases following a multimodal approach combining bilateral cortical rTMS, transcutaneous electrical nerve stimulation (TENS), and cricopharyngeal myotomy [55]. Notably, this study reported only two cases that received combined treatments, making it unclear which treatment, or combination of treatments, produced the effect. In an earlier randomized trial, Khedr and Abo-Elfetoh [55] observed improvements in swallowing scores in brainstem infarction patients treated with bilateral low-frequency (3 Hz) rTMS applied over bilateral esophageal motor cortices. Most recently, Wu et al. [58] conducted a large-scale network meta-analysis including patients with infratentorial strokes, concluding that high-frequency bilateral cerebellar rTMS and combined hemispheric stimulation protocols were among the most effective, especially in the acute and subacute phases of stroke recovery. Collectively, these studies strengthen the case for using rTMS to engage preserved cortical and cerebellar circuits in the rehabilitation of swallowing function after brainstem injury.

In summary, the accumulated evidence suggests that rTMS is a safe, non-invasive, and potentially effective therapy for dysphagia rehabilitation in brainstem stroke. Given the centrality of the medulla in swallowing control and the poor response to traditional interventions in severe cases, rTMS offers a novel therapeutic angle, particularly when used early and in combination with behavioural therapy. The interaction between cortical stimulation and peripheral sensory feedback (e.g., via fibreoptic endoscopic evaluation of swallowing [FEES] or behavioural therapy) likely enhances reorganization in distributed swallowing networks, implying that combining rTMS with standard swallowing exercises may yield better outcomes than either modality alone. While rTMS for brainstem stroke induced dysphagia is still evolving, its neurophysiological rationale is robust, and early-phase studies indicate clinically meaningful improvements, especially in carefully selected patients. Further multicentre trials and individualized stimulation protocols are needed to fully define its role in clinical practice.

### 3.3. Transcranial Direct Current Stimulation (tDCS)

Transcranial direct current stimulation (tDCS) is another form of NIBS technique that can modulate neuronal depolarization thresholds and induce N-methyl-D-aspartate (NMDA)-mediated neuroplasticity changes by delivering weak electric current onto the brain through surface electrodes placed on the scalp [66,67,68,69,70].

Studies on the effects of tDCS in modulating swallowing neural network focused on its application on the motor cortex. Depending on the mode of stimulation, tDCS can induce long-lasting increase (anodal tDCS) or decrease (cathodal tDCS) in the excitability of the pharyngeal motor cortex [30]. In healthy individuals, anodal tDCS can enhance the processing efficiency of swallowing neural networks [71], excitability of suprahyoid motor cortex [72], and swallowing function and biomechanics [71,73]. Furthermore, when the swallowing neural network is disrupted by rTMS-induced “virtual lesion”, anodal tDCS can reverse the neurophysiological effects [74,75].

Several recent meta-analyses suggested that tDCS can improve swallowing function, reduce dysphagia severity and risks of aspiration in patients with post-stroke dysphagia [38,49,76,77,78,79,80]. However, there is a substantial heterogeneity in study protocols, patient characteristics and tDCS stimulation parameters among the RCTs in the literature. Most studies investigated the short-term (within 2 weeks) effects of anodal tDCS, such that the long term (beyond 6 months) maintenance effects of tDCS remain unknown [38]. Moreover, the stimulation hemisphere for optimal outcomes is under debate, with contradictory conclusions from meta-analyses. A meta-analysis revealed that contralesional stimulation yielded significant treatment effects, but bihemispheric stimulation did not [38], while another meta-analysis suggested that bihemispheric stimulation showed a stronger effect than unihemispheric stimulation [76].

Given that most RCTs included a mixture of patients with various stroke locations, it is difficult to isolate the effects for patients with brainstem stroke. Nonetheless, a meta-analysis by Zhao et al. [79] found that tDCS was effective for dysphagia after brainstem stroke, unilateral hemispheric stroke, and bulbar paralysis, but not after ataxic and basal ganglia stroke. However, this finding could be because only one RCT was available for analysis for ataxic and basal ganglia stroke patients.

Some recent RCTs on the effects of tDCS included patients with brainstem stroke (Table 2). Mao et al. [63] randomized 40 patients with brainstem stroke into receiving real or sham anodal tDCS with swallowing rehabilitation training. The anodal tDCS was delivered at 1.6 mA for 20 min per day for 54 days over the swallowing sensorimotor cortex. The active tDCS group showed greater improvement in swallowing function and nutritional indexes than the sham group. In another study by Wang et al. [61], twenty-eight brainstem stroke patients with cricopharyngeal muscle dysfunction (CPD) were randomized to receive real or sham anodal tDCS overall bilateral esophageal motor cortex with simultaneous catheter balloon dilation and conventional swallowing treatments for 20 days. They found that anodal tDCS improved swallowing function, as measured by FDS and FOIS scores, and pharyngoesophageal segment opening (PES) function. Other studies have included brainstem stroke patients as part of their sample, but separate analysis on the effects among them were not reported (Table 2).

Although tDCS is a safe technique with potential benefits for post-stroke dysphagia, the evidence on its clinical efficacy specifically for patients with brainstem stroke remains controversial. Given that tDCS modulates the threshold of membrane depolarization instead of directly depolarizes the nerve cells, its effects on the swallowing system may need to be complemented with afferent stimulation or behavioural approaches to achieve optimal outcomes.

***Limitations of neuromodulation.*** While the evidence for neuromodulatory treatments appears promising, their use in the stroke population comes with limitations. First, evidence from brainstem stroke-specific studies remains sparse and often underpowered. Safety data are limited for patients with more extensive or bilateral lesions. Second, the heterogeneity in stimulation parameters (e.g., frequency, intensity, duration) and outcome measures impede protocol standardization. Importantly, the optimal stimulation site for NIBS techniques—whether cortical, contralesional, or cerebellar—remains under debate. Additionally, while cerebellar rTMS has shown cortical excitability enhancement, not all studies have translated this into clear functional gains [50]. Moreover, variability in the response to NIBS due to individual genetic predisposition [81], anatomical differences, or comorbidities may affect the treatment efficacy. Nonetheless, recent study suggested that such variability may be overcome by targeted or preconditioned neuromodulatory approach [82]. Finally, while NIBS protocols can manipulate cortical inputs and promote swallowing recovery through inducing targeted neuroplastic changes in the swallowing neural network, they do not target specific biomechanical or pathophysiological features of swallowing.

In summary, neuromodulatory techniques such as PES, rTMS and tDCS have potential therapeutic value in improving swallowing function following brainstem stroke. Nonetheless, the evidence on the clinical efficacy in this population remains limited. As such, clinical guidelines on the application of PES, rTMS and tDCS tailored to patients with brainstem stroke are not yet available. For further insights into the safety recommendations and clinical guidelines, readers can refer to the relevant publications [83,84,85]. Further studies with careful documentation and patient selection may elucidate the roles of neuromodulatory techniques in clinical practice.

## 4. Skill-Based Swallowing Training

The shortcomings of NIBS techniques provide justification for additional behavioural rehabilitation approaches. Our behavioural rehabilitation approaches in general have evolved considerably as a result of increased understanding of neural control of swallowing. Early research by Jean, Miller, and colleagues identified a brainstem-driven CPG as the central control mechanism for swallowing, initially excluding significant cortical involvement [86,87,88,89,90]. Behavioural management in the early days was consistent with our knowledge at that time. As swallowing was viewed largely as a reflex, early dysphagia management practices focused on compensatory strategies. Later, muscle strengthening approaches were implemented with the idea that increasing force generation though exercise may facilitate improved bolus flow [91,92]. Initially nonspecific, these exercises have become more targeted to weakness of specific muscle groups or biomechanical deficits [93,94,95]. These approaches have predominantly persisted despite advances in our understanding of swallowing motor control.

Very early, Martin and Sessle [96] emphasized the importance of cortical input for volitional swallowing. Advances in neuroimaging have since highlighted the role of both cortical and subcortical structures in swallowing motor control. Importantly, Ertekin [97] and Mosier & Bereznaya [98] proposed models integrating sensory and motor cortices with the medullary CPG, very importantly offering a distinction between reflexive and volitional swallowing behaviours. Recent thinking has shifted terminology from ‘swallowing reflex’ to ‘pharyngeal swallowing response,’ underscoring the importance of cortical modulation in ingestive behaviour [31,99,100]. The extent to which cortical networks modify or augment the medullary CPG remains an open question.

As discussed, NIBS may promote swallowing recovery by targeted neuroplastic change, yet the effects are non-specific. More focused stimulation or pairing it with behavioural activation of task-related cortical circuits might yield better, more stable outcomes. However, this will only be the case if behavioural approaches are task specific. Priming cortical motor networks to modulate excitability before motor training could also enhance rehabilitation potential [101]. This raises critical questions: Should cortical stimulation be paired with peripheral strengthening exercises? Or, will an approach of swallowing skill be more appropriate to effectively recruit central neural mechanisms?

Early in the application of muscle strengthening, the use of surface electromyography (sEMG) biofeedback modalities was incorporated into practice. Bryant’s 1991 case study [102] introduced sEMG as a biofeedback tool for mastery of the effortful swallow and Mendelsohn manoeuvre in a head and neck cancer patient. Further clinical case series reported positive swallowing outcomes in patients with brainstem injury following intensive rehabilitation programmes utilising sEMG biofeedback [92,103] and in the general stroke population [104]. The use of sEMG biofeedback in swallowing rehabilitation has more recently been the focus of two systematic reviews [105,106]. These reviews suggest a few modest changes in swallowing parameters, but importantly, they highlight significant questions. What is the active treatment when using sEMG for swallowing rehabilitation? Did patients recover, or fail to recover, due to peripheral muscle strengthening exercises? Or was change in function facilitated, or inhibited, from the modulation of swallowing behaviour arising from visualization and adaptation of movement—the concept of swallowing skill?

Swallowing skill training as a therapeutic approach has arisen from the recognition of cortical contributions to swallowing and has been enhanced by our understanding of neuroplasticity and its application to swallowing management. In a 1972 publication that preceded much of the foundational research on the swallowing CPG [1,2,3,4,5], Larsen [107] introduced the concept of using cortical input to enhance swallowing performance. He stated “*<the patient> is taught the importance of regulating his swallowing volitionally rather than on a reflex basis. In other words, swallowing is made subject to intellectual control…. He will be taught to “think swallow” and then swallow.*” (pp. 189–190). Furthermore, Kleim and Jones [108] define neural plasticity as the brain’s mechanism for encoding experience and learning new behaviours, including relearning lost behaviours post-damage. Their work outlines ten principles of experience-dependent plasticity which ultimately provide a strong foundation for the development of skill-based training approaches. Robbins et al. [109] conceptually applied these principles to swallowing, discussing key strategies for integrating neuroplasticity into practice. Recognizing cortical modulation and neuroplasticity opens new avenues for swallowing rehabilitation, particularly for those whose dysphagia is not due to muscle weakness.

Research in healthy individuals has started to investigate the relationship between cortical focus and swallowing neural control. Jing et al. [110] found that engaging perceptual and cognitive schemes of swallowing activates specific neural networks, as shown by fMRI studies. Both actual and imagined swallowing activated the supplementary motor area (SMA) and left middle temporal gyrus, indicating potential for cortical reorganization. Kober et al. [111] confirmed these findings and showed that neurofeedback could further enhance activation in targeted and additional cortical regions. Translating these principles to rehabilitation approaches, Szynkiewicz et al. [112] demonstrated that a 6-week mental practice regime, where participants imagined lingual strengthening exercises, significantly improved lingual strength. These studies provide preliminary findings that support the role of cortical control and feedback in swallowing rehabilitation.

Outcome studies of skill training are slowly emerging, but with few examples in brainstem stroke (Table 3). Athukorala et al. [113] applied sEMG biofeedback to skill-based training, improving precision in submental muscle contraction timing and magnitude. Ten patients with Parkinson’s disease completed 10 h of skill training over two weeks, hitting randomly placed targets on a computer screen with calibrated non-effortful swallowing. Skill was required to predict placement of swallowing sEMG peak in both timing and strength domains. Significant improvements were noted in functional swallowing measures, sEMG activity, and swallowing-related quality of life [114,115]. Training with saliva swallowing showed transference to liquid bolus swallowing, indicating skill acquisition. A number of other small studies have applied similar protocols in patients with varied aetiologies [116,117] although with none focusing predominantly on brainstem injury. Three of these were small randomized controlled trials [118,119,120].

A more recent case series in the swallowing skill literature, one of the only studies in patients with brainstem injury reported on the use of low-resolution pharyngeal manometry to modulate pressure patterns in swallowing. Huckabee et al. [121] reported on a cohort of 16 patients, all with infratentorial stroke or brain tumour resection, and all with atypical pharyngeal pressure generation, characterized by no superior to inferior pressure wave. Patients were coached to increase temporal separation of pressure peaks. Twelve of 16 patients, who were able to participate in daily treatment for a minimum of two weeks, returned to normal oral diet, with resolution of nasal redirection, aspiration, and pharyngeal residue.

***Limitations of skill-based swallowing training:*** Despite the understanding of cortical involvement in swallowing and neuroplasticity, the construct of swallowing skill training as a behavioural rehabilitation approach is still in early development. Further critical work is needed on specifics of application, intended biomechanical or pathophysiological targets, and clinical outcomes. Several theoretical articles on the topic are available to interested readers [122,123,124]. Moreover, clinical trials focus on patients with dysphagia brainstem stroke are warranted to elucidate the treatment effects in this population.

Although the effects of skill-based swallowing training remain uncertain for patients with brainstem lesions due to limited data, clinical decisions may be guided by the pathophysiology of swallowing impairment. For instance, it is quite reasonable to assume that patients with brainstem lesions may well present with isolated or partial strength impairment due to lower motor neuron involvement. In this case, the more traditional strength training approaches remain appropriate. However, for those with nuclear or supranuclear involvement, impaired motor planning or execution may be inhibiting efficient swallowing, thus requiring the emerging approach of skill training.

## 5. Transient Receptors Potential (TRP) Agonists

Sensory inputs are vital for triggering of swallowing and modulating motor swallowing response [96,125]. The oropharynx contains numerous TRP channels that provide sensory information of the food bolus as it passes along the swallowing tract [96,126]. TRP channels, including transient receptor potential vanilloid 1 (TRPV1), transient receptor potential ankyrin 1 (TRPA1), and transient receptor potential melastatin 8 (TRPM8), are sensitive to a range of temperature and chemicals [127,128,129,130,131]. When these channels are activated, depolarization of sensory neurons leads to triggering of sensory impulses, which are then transmitted to the NTS of the medulla and the sensorimotor cortex through cranial nerves [96,125].

Given the importance of sensory input in swallowing, several peripheral stimulation treatments, for example, thermal, chemical or mechanical stimulation, have been investigated to enhance swallowing in patients with dysphagia. Among these, TRP channel agonists have been most extensively studied, likely due to the abundance of TRP channels in the oropharynx and the variety of natural TRP agonists that can be used to activate them, and they showed the greatest therapeutic potential in patients with post-stroke dysphagia [132,133].

Studies showed that TRP agonists may improve swallowing through increasing the secretion of salivary neuropeptides, in particular substance P and calcitonin gene-related peptide (CGRP) [134,135]. In patients with post-stroke dysphagia, reduced levels of these neuropeptides have been linked to increased pharyngeal sensory thresholds [136] and reduced spontaneous swallowing frequency [137], a factor correlated with increased disability, higher rates of institutionalization, and mortality after stroke [138]. The increase in these neuropeptides induced by TRP agonists may enhance sensory perception and promote faster transmission of afferent sensory signals to the brainstem, leading to improved swallowing function. Importantly, the effects of TRP agonist are dose-dependent, with low concentration and single application eliciting short-term facilitatory effect on sensory neurons, while repeated applications induce changes in the event-related potentials in cortical regions such as cingulate gyrus and the medial frontal gyrus [135,139].

In patients with post-stroke dysphagia, evidence supports the therapeutic effects of various natural TRP agonists on the timing of the oropharyngeal swallow response [139,140,141,142] (Table 4). Studies showed that acute oral stimulation with capsaicin (a TRPV1 agonist, 150 µM), piperine (a TRPA1/V1 agonist, 150 µM and 1 mM), cinnamaldehyde-zinc (a TRPA1 agonist, 100 ppm–70 mM), and citral (a TRPA1 agonist, 250 ppm) significantly reduced the time to laryngeal vestibule closure (LVC) by approximately 100 ms [139,140,141,142]. Furthermore, stimulation with TRPV1 and selective TRPA1 agonists could reduce the time to upper esophageal sphincter opening (UESO) by around 70 ms and increase both the pharyngeal contractile integral and the duration of upper esophageal sphincter activation and relaxation [139,140,141,142]. Among the tested compounds, TRPA1/V1 agonists demonstrated the greatest potential for reducing the prevalence of unsafe swallows by up to 50%. Additionally, a study by Tomsen et al. found that stimulation with capsaicin and piperine at 150 µM significantly increased bolus velocity [143]. Regarding the effects on spontaneous swallowing, studies showed that direct administration of capsaicin into the pharynx of post-stroke patients reduced the latency of the swallowing response and enhanced the cough reflex [144]. Nascimento et al. found that oral administration of four 10 mL boluses of capsaicin at 10 µM significantly increased the spontaneous swallowing frequency by 50% in patients with post-stroke dysphagia, without affecting the electromyographic activity of the suprahyoid muscles [145]. Furthermore, a randomized controlled trial by Wang et al. demonstrated that oral capsaicin improved swallowing function in patients with post-stroke dysphagia [146].

***Limitations of TRP agonists:*** Although preliminary research suggests that TRPV1 agonists may improve swallowing function in patients with post-stroke dysphagia the application of these compounds remains in the realm of clinical research and not of clinical translations. Similar to the other treatment options discussed, most studies have focused on patients with mixed stroke lesions, with limited data specifically addressing dysphagia following brainstem stroke. This evidence gap is critical, as brainstem strokes may involve damage to the sensory and/or motor neural pathways, which could influence the swallowing system’s response to sensory stimulation by TRP agonists. Furthermore, available clinical studies lack detailed descriptions of patient characteristics, such as lesion location and stroke severity, making it difficult to delineate treatment effects in patients with brainstem stroke. Therefore, future clinical trials are needed to investigate the therapeutic potential, optimal dosing, and long-term outcomes of TRP agonists in patients with dysphagia following brainstem stroke.

### Future Directions

The management of dysphagia following brainstem stroke remains a complex challenge that warrants further research. This review highlights several research directions for future investigations. First, multicentre, adequately powered clinical trials that stratify by brainstem lesion site and laterality should be conducted to evaluate the treatment benefits in this population. Furthermore, given the complex and unique complications of dysphagia following brainstem stroke, it is essential to investigate both short- and long-term outcomes in this population. Finally, future studies should explore the potential of combined treatment, or individualized treatment guided by biomarker or neuroimaging findings to address the limitations of existing treatment options. Until such data become available, clinicians should exercise caution when applying these emerging treatments, recognizing that the current recommendations are primarily based on extrapolated evidence rather than findings specific to brainstem stroke.

## 6. Conclusions

The brainstem plays an important role in coordinating the swallowing process. Brainstem stroke may damage both sensory inputs and motor outputs, leading to severe and persistent dysphagia characterized by delayed or absent swallowing reflexes and aspiration. Recent advances in neuromodulation, skill-based swallowing training and TRP agonist treatment provide promising rehabilitation options for these patients. Nonetheless, the current evidence on clinical efficacy specific to this population remains scarce. Given this limitation, management decisions should rely on the principles of swallowing physiology, neuroplasticity and clinical findings from other stroke populations. This narrative review highlights the need for multicentre studies focusing on the brainstem-lesioned population to investigate short- and long-term effects on clinical and functional outcomes. Moreover, future studies may explore the value of individualized treatment, combined neuromodulatory and behavioural intervention, and optimized protocols to enhance treatment outcomes.

## Figures and Tables

**Table 1:** Key components of the swallowing network in the brainstem and the relevant dysphagia symptoms.

Structure	Location	Role	Function	Symptoms After Lesion
**Nucleus Tractus Solitarius (NTS)**	Medulla	Sensory Centre	Main sensory nucleus Receives swallowing-related sensory input Regulates swallowing reflex	Difficulty initiating swallowing Aspiration Delayed swallowing reflex
**Nucleus Ambiguus (NA)**	Medulla	Motor Centre	Controls muscles of the pharynx, larynx, and upper esophagus Coordinates muscle activity during swallowing	Difficulty swallowing Aspiration Airway obstruction
**Central pattern generator (CPG)**	Medulla	Coordination Centre	Coordinates muscle activity for swallowing Ensures rhythmic and coordinated swallowing	Disrupted swallowing rhythm Difficulty coordinating swallowing stages
**Cranial Nerve Nuclei**	Pons, Medulla	Swallowing Regulator	Controls muscle movements in the oral cavity, pharynx, and larynx Regulates sensory and motor aspects of swallowing	Oral-phase swallowing difficulty Tongue movement problems Aspiration

**Table 2:** Summary of repetitive transcranial magnetic stimulation (rTMS) and transcranial direct current stimulation (tDCS) studies in patients with dysphagia following brainstem stroke.

Study	Design	Population	Stimulation Parameters	Outcomes	Limitations
**rTMS**					
Khedr & Abo-Elfetoh, 2010 [54]	RCT	22 patients with brainstem infarction and LMS	3 Hz, bilateral hemisphere, 10 min/day, 5 days	Improved swallowing severity (DG score)	No blinding; limited sample
Verin et al., 2016 [55]	Case series	2 patients with chronic aphagia post-LMS	1 Hz, bilateral motor cortex, 20% above threshold, 5 × 5 days + TENS + surgery	Full restoration of oral intake	Small sample; multimodal approach limits causal inference
Lin et al., 2018 [56]	Proof-of-concept	28 patients with brainstem stroke with dysphagia	Vagal magnetic modulation, 600 pulses/day, 10 days	Significant recovery in AusTOMs swallowing domain	Non-TMS coil; vagus targeting—limited generalizability
Dong et al., 2022 [50]	RCT	34 patients with medullary/pontine stroke	10 Hz, 250 pulses, 80% RMT, bilateral/unilateral cerebellum vs. sham, 2 weeks	Improved PAS & FDS scores; increased MEP amplitudes	No direct correlation between MEP gain & clinical improvement
Dai et al., 2023 [57]	RCT (single-blinded)	42 subacute infratentorial stroke patients	10 Hz, 5 × 50 stimuli, 90% RMT, bilateral/unilateral cerebellum vs. sham, 10 days	Significant FOIS, PAS, DOSS improvements; Bilateral > unilateral	No significant MEP differences; cerebellar lesion variability may affect results
Wu et al., 2024 [58]	Network meta-analysis	760 PSD patients (including brainstem stroke)	Multiple protocols including HF/ipsi-CRB, HF/bi-CRB	HF/bi-CRB, HF/ipsi-CRB improved swallowing (PAS, FDS)	Protocol heterogeneity; brainstem subgroup effects not isolated
**tDCS**					
Shigematsu et al., 2013 [59]	RCT	20 stroke patients (7 with brainstem stroke)	1 mA, 20 min, 10 days; Ipsilesional pharyngeal motor cortex	Improved DOSS	Unclear which side was targeted for brainstem stroke patients
Suntrup-Krueger et al., 2018 [60]	RCT	59 stroke patients (14 with brainstem stroke)	1 mA, 20 min, 4 days; Swallowing (pharyngeal) motor cortex; Right hemisphere for brainstem stroke	Improved FEDSS; associated with increase in activation of contralesional swallowing neural network	Brainstem subgroup effects not isolated
Wang et al., 2020 [61]	RCT	28 patients with brainstem stroke and CPD	1 mA, 20 min, 20 days; Bilateral oesophageal motor cortex	Improved FDS and FOIS; Improved PESO scores	Unclear methodology: one anodal electrode for bilateral stimulation; Sequence of hemispheric stimulation unclear
Farpour et al., 2022 [62]	RCT	44 stroke patients (2 with brainstem stroke, both received active tDCS)	2 mA, 20 min, 5 days; Supramarginal gyrus; Right hemisphere for brainstem stroke	Improved MASA and FOIS	No patients with brainstem stroke in the sham group
Mao et al., 2022 [63]	RCT	40 patients with brainstem stroke	1.6 mA, 20 min, 54 days; Unlesioned swallowing sensory motor cortex	Improved DOSS and FDS; Improved nutritional indexes	Unclear which hemisphere was targeted for brainstem stroke patients

AusTOMs: Australian Therapy Outcome Measures; CRB: cerebellum; CPD: cricopharyngeal muscle dysfunction; DG: dysphagic grade; DOSS: Dysphagia Outcome and Severity Scale; FEDSS: Fiberoptic Endoscopic Dysphagia Severity Scale; FOIS: Functional Oral Intake Scale FDS: Functional Dysphagia Scale; LMS: lateral medullary syndrome; MEP: motor evoked potential; PAS: Penetration Aspiration Scale; PESO: pharyngoesophageal segment opening; PSD: post-stroke dysphagia; RMT: resting motor threshold; TENS: transcutaneous electrical nerve stimulation.

**Table 3:** Clinical studies on skill-based swallowing training.

Study	Design	Population	Skill Training Protocol	Biofeedback	Outcomes
Athukorala et al. [113]	Observational	10 patients with PD	Skill training targeted at improving strength and timing of swallowing movements. 10 sessions over 2 weeks	sEMG activity of submental muscles with sEMG activity displayed on a computer monitor	Improved functional swallowing measures, sEMG activity, and swallowing-related quality of life
Battel & Walshe, 2023 [117]	Observational	10 patients with PD	Skill training targeted at coordinating swallowing and increasing submental muscle activity. 5 days a week for 4 weeks	sEMG activity of submental muscles with visualization of sEMG activity through a computer game	Improved oral intake methods and in pharyngeal residue from saliva and solids.
Benfield et al., 2023 [119]	RCT on feasibility	27 patients with acute (≤4 weeks) * post-stroke dysphagia	Experimental group: CDT + sEMG-BF training; Skill training targeted at improving strength and timing of swallowing movements. Control: CDT 10 sessions over 2 weeks	sEMG activity of submental muscles with sEMG activity displayed on a computer monitor	The treatment protocol is feasible with compliance rate of 80%.
Hou et al., 2024 [120]	RCT	90 patients with acute (≤2 weeks) post-stroke dysphagia (25 with brainstem stroke)	Experimental group 1: tDCS + sEMG-BF + NMES + CDT; Skill training targeted at performing effortful swallow. Experimental group 2: tDCS + sEMG-BF + game training + CDT; Skill training targeted at performing Mendelsohn maneuver. Control: tDCS + CDT All treatments were delivered for 20 min per day for 7–14 days.	Group 1: sEMG activity of submental muscles Group 2: sEMG activity of submental muscles with visualization of sEMG activity through a computer game	Improved functional swallowing measures, sEMG activity, swallowing timing and tongue pressure in both experimental groups. Game training combined with biofeedback showed the greatest improvement among the three groups.
Huckabee et al., 2014 [121]	Observational	16 patients with infratentorial stroke or brain tumour resection, and all with atypical pharyngeal pressure generation	Skill training targeted at increasing the temporal separation between the upper and lower pharyngeal pressure waveforms when swallowing. Twice daily for a minimum of one week.	Manometric measurement of the pharynx with visualization of pharyngeal pressure displayed the manometric system	12 patients returned to normal oral diet, with resolution of nasal redirection, aspiration, and pharyngeal residue.
Nordio et al., 2022 [118]	RCT	16 patients with post-stroke (>6 weeks) dysphagia (12 with brainstem stroke)	Experimental group: sEMG-BF rehabilitation; Skill training targeted at performing effortful swallow, supraglottic swallow and Masako maneuver. Control: Behavioural training without sEMG-BF. All treatments were delivered for 1 h per day for 5 days	sEMG activity of submental muscles with sEMG activity displayed on a computer monitor	sEMG-BF improved pharyngeal clearance and swallowing safety compared to control.
Perry et al., 2018 [116]	Case study	1 patient with multiple system atrophy	Skill training targeted at improving strength and timing of swallowing movements. 6 sessions over 6 weeks + daily home practice	sEMG activity of submental muscles with sEMG activity displayed on a computer monitor	Improved accuracy in swallowing movements; reduced premature spillage and aspiration and post-swallow residue; subjective improvement in swallowing symptoms

CDT: conventional dysphagia treatment; NMES: neuromuscular electrical stimulation; RCT: randomized controlled trial; sEMG: surface electromyography; sEMG-BF: surface electromyography with biofeedback; tDCS: transcranial direct current stimulation; * Location of stroke not specified.

**Table 4:** Clinical studies on Transient Receptor Potential (TRP) agonists.

Study	Design	Population	Treatment Protocol	Outcomes
Ebihara et al., 2006 [144]	RCT	67 patients with * post-stroke dysphagia	Nasal inhalation of black pepper oil (concentration unspecified) vs. lavender oil vs. distilled water. 1 min before each meal for 30 days	Improved latent time of swallowing reflex, increased serum substance P level, increased number of involuntary swallowing movements during nasal inhalation of black pepper oil.
Nascimento et al., 2021 [145]	Observational	141 healthy volunteers and 17 patients with * post-stroke dysphagia	10 μM oral capsaicin	Capsaicin increased spontaneous swallowing frequency when comparing to basal condition.
Rofes et al., 2013 [141]	Observational	33 patients with neurogenic dysphagia	150 μM capsaicinoid (oral)	Treatment with capsaicinoids reduced penetration and pharyngeal residue, shortened the time of laryngeal vestibule closure, upper esophageal sphincter opening, and maximal hyoid and laryngeal displacement
Rofes et al., 2014 [140]	RCT with active control	40 elderly with dysphagia associated with ageing, non-progressive neurological disease or neurodegenerative disease	150 μM piperine (oral) vs. 1 mM piperine (oral)	Improved swallowing safety and reduced laryngeal vestibule closure time. Greater effects observed at higher concentration.
Tomsen et al., 2019 [139]	RCT	14 elderly with dysphagia associated with ageing	(a) 10 mL of 10 μM capsaicin (oral) vs. placebo; Single dose (b) 10 mL of 10 μM capsaicin (oral) vs. placebo; 10 days	The 10-day treatment regimen induced cortical changes that were correlated with reduced laryngeal vestibule closure time and aspiration and penetration in older patients with dysphagia.
Tomsen et al., 2022 [143]	Retrospective	329 patients with dysphagia	Oral capsaicin (TRPV1, 150 μM/10 μM), piperine (TRPA1/V1, 1 mM/150 μM), menthol (TRPM8, 1 mM/10 mM), cinnamaldehyde-zinc (TRPA1, 100 ppm–70 mM), citral (TRPA1, 250 ppm) and citral-isopulegol (TRPA1-TRPM8, 250–200 ppm)	Capsaicin 150 μM or piperine 1 mM significantly improved swallowing safety and time of laryngeal vestibule closure and bolus velocity.
Wang et al., 2019 [146]	RCT	60 patients with post-stroke dysphagia (12 with brainstem or cerebellar stroke)	150 μM/L capsaicin (oral) (thermal tactile stimulation + nectar bolus). 3 times per day for 21 days	Improved swallowing function as assessed by Eating Assessment Tool [147] and Standardized Swallowing Assessment

RCT: randomized controlled trial; TRPA1: transient receptor potential ankyrin 1 (TRPA1); TRPV1: transient receptor potential vanilloid 1; TRPM8: transient receptor potential melastatin 8; * Location of stroke not specified.

## Data Availability

Data sharing is not applicable.

## Abbreviations

AusTOMs	Australian Therapy Outcome Measures
CRB	Cerebellum
CGRP	Calcitonin gene-related peptide
CPD	Cricopharyngeal muscle dysfunction
CPG	Central pattern generator
CT	Computerized topography
DG	Dysphagic grade
DOSS	Dysphagia Outcome and Severity Scale
DSG	Dorsal swallowing group
EC	European Commission
FDA	Food and Drug Administration
FDS	Functional Dysphagia Scale
FEES	Fibreoptic endoscopic evaluation of swallowing
fMRI	Functional magnetic resonance imaging
FOIS	Functional Oral Intake Scale
LMS	Lateral medullary syndrome
LVC	Laryngeal vestibule closure
MEP	Motor evoked potential
MRI	Magnetic resonance imaging
NA	Nucleus ambiguus
NIBS	Non-invasive brain stimulation
NMDA	N-methyl-D-aspartate
NTS	Nucleus tractus solitarius
PAS	Penetration Aspiration Scale
PES	Pharyngeal electrical stimulation
PSD	Post-stroke dysphagia
RCT	randomized controlled trial
RMT	Resting motor threshold
RTMS	Repetitive transcranial magnetic stimulation
SMA	supplementary motor area
SMD	standardized mean difference
sEMG	Surface electromyography
TENS	Transcutaneous electrical nerve stimulation
TDCS	Transcranial direct current stimulation
TRP	Transient receptors potential
TRPA1	Transient receptor potential vanilloid 1
TRPM8	Transient receptor potential ankyrin 1
TRPV1	Transient receptor potential melastatin 8
UESO	Upper esophageal sphincter opening
VSG	Ventral swallowing group
